# Delayed urticaria after the third dose of mRNA COVID19 vaccine: A case series

**DOI:** 10.1111/dth.15680

**Published:** 2022-07-11

**Authors:** Leonardo Bianchi, Katharina Hansel, Filippo Biondi, Maddalena Napolitano, Cataldo Patruno, Gabriella Fabbrocini, Luca Penchini, Luca Stingeni

**Affiliations:** ^1^ Section of Dermatology, Department of Medicine and Surgery University of Perugia Perugia Italy; ^2^ Section of Dermatology, Department of Clinical Medicine and Surgery University of Naples Federico II Naples Italy; ^3^ Section of Dermatology, Health Sciences Department Magna Graecia University Catanzaro Italy

Dear Editor,

Drug hypersensitivity reactions can be immediate (within 1–6 h after drug exposition), or delayed (at any time as from 1 h after drug administration).^1^ Among the latter, drug‐related delayed urticaria occurs from 4 h to several days after drug intake,^1^ but this nosological entity is not systematically reported in position papers.^2^ The implementation of massive vaccination campaigns to COVID19 infection has increased the attention to COVID19 vaccine hypersensitivity reactions, especially for immediate ones and rarely for delayed ones. Recently, some cases of delayed urticaria were reported after both the first and second COVID19 vaccine doses,^3^, ^4^, ^5^, ^6^, ^7^ mostly after Pfizer‐BioNTech BNT162b2 and Moderna mRNA‐1273.

Patients with delayed urticaria occurring at least 4 h after the third dose of mRNA COVID19 vaccine during a 2 month period (January and February 2022) and who well tolerated the first and second vaccine dose, were prospectively collected. The study was approved by the ethic committees of the Perugia, Naples, and Catanzaro Universities and informed consent was obtained from all participants for both study participation and publication. All patients were evaluated according to the international guidelines for urticaria.^8^ Twenty‐three patients developed urticaria from 2 to 21 days (mean latency time: 9.7 days) after the third dose of COVID19 mRNA vaccine with marked dermographism and intense itch (Figure 1), associated to angioedema in 5 (21.8%) but never to anaphylaxis. Demographics and clinical data of patients and characteristics of vaccine‐induced delayed urticaria are reported in Table 1. Two patients (8.7%) suffered from acute urticaria in the past and three suffered from autoimmune thyroiditis, two of them in euthyroidism. Twenty‐one (91.3%) developed delayed urticaria after Moderna mRNA‐1273 vaccine and the remaining 2 after Pfizer‐BioNTech BNT162b2. Thirteen patients (56.5%) were treated with oral corticosteroids in addition to antihistamines; resolution mean time was 10 days (range 5–20 days) in 21 subjects while in patient n.12 and n.13 urticaria became chronic.

**FIGURE 1:**
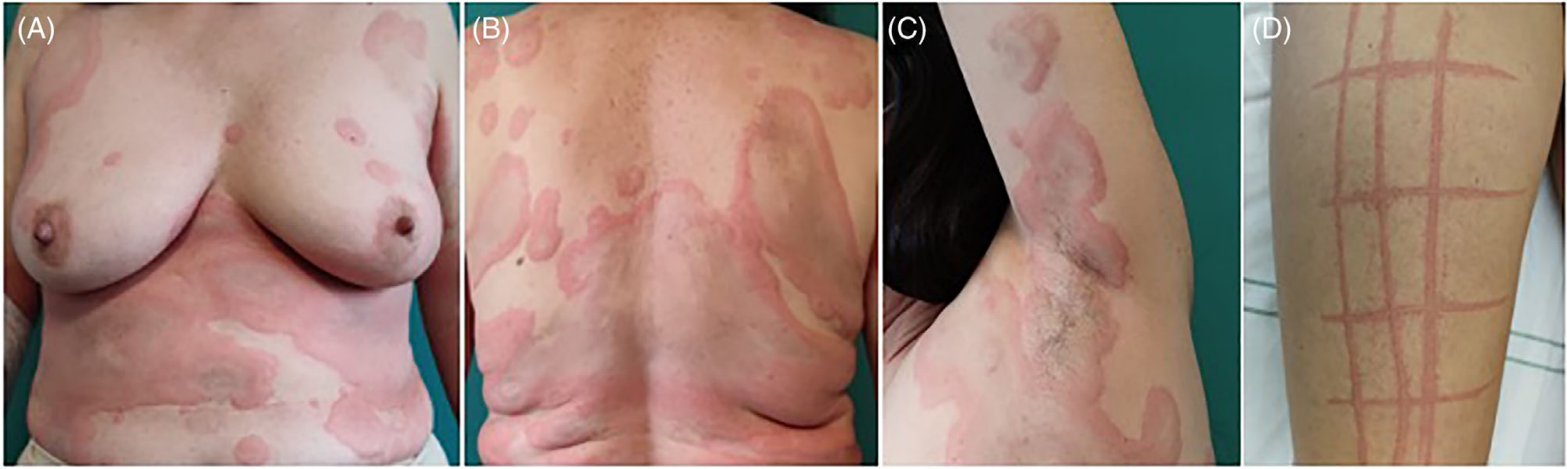
Urticaria with wide and confluent wheals on trunk (A, B) and upper limb (C) with Marked dermographism (D) in patient n. 11 (latency time: 10 days after the third dose of mRNA‐1273 COVID19 vaccine)

**TABLE 1:** Demographics and clinical data of patients and characteristics of vaccine‐induced delayed urticaria

Patient n.	Sex, age	Atopy	Previous urticaria	Concomitant diseases	Vaccine	Latency (days)	Angio‐edema	Treatment	Resolution (days)
1	M, 33	‐	Yes	Autoimmune thyroiditis	mRNA‐1273	10	No	Antihistamines	10
2	F, 25	‐	No	‐	mRNA‐1273	21	Yes	Antihistamines	5
3	F, 22	‐	No	‐	mRNA‐1273	10	Yes	Antihistamines	7
4	M, 28	RC	Yes	‐	mRNA‐1273	8	Yes	Antihistamines	6
5	F, 22	‐	No	‐	mRNA‐1273	11	No	Antihistamines	10
6	M, 30	RC	No	‐	mRNA‐1273	10	No	Antihistamines	6
7	F, 26	RC	No	Autoimmune thyroiditis	mRNA‐1273	10	No	Antihistamines, corticosteroids	4
8	M, 27	RC	No	‐	mRNA‐1273	11	No	Antihistamines	5
9	F, 22	RC	No	‐	mRNA‐1273	10	Yes	Antihistamines	8
10	M, 28	‐	No	‐	mRNA‐1273	10	No	Antihistamines, corticosteroids	10
11	F, 38	RC	No	‐	mRNA‐1273	10	No	Antihistamines	13
12	F, 67	‐	No	Hypertension	mRNA‐1273	11	No	Antihistamines, corticosteroids	Chronic urticaria
13	F, 28	RC	No	Autoimmune thyroiditis	mRNA‐1273	2	Yes	Antihistamines, corticosteroids	Chronic urticaria
14	F, 33	‐	No	‐	mRNA‐1273	9	No	Antihistamines, corticosteroids	11
15	M, 32	RC	No	‐	mRNA‐1273	10	No	Antihistamines, corticosteroids	10
16	F, 34	RC, AD	No	‐	mRNA‐1273	10	No	Antihistamines	10
17	F, 36	RC	No	‐	BNT162b2	10	No	Antihistamines, corticosteroids	15
18	M, 22	RC, AD	No	‐	mRNA‐1273	7	No	Antihistamines, corticosteroids	15
19	M, 48	RC, AD	No	Hypertension	mRNA‐1273	8	No	Antihistamines, corticosteroids	10
20	F, 39	AD	No	‐	mRNA‐1273	10	No	Antihistamines, corticosteroids	20
21	M, 56	‐	No	‐	mRNA‐1273	7	No	Antihistamines, corticosteroids	15
22	M, 29	RC	No	‐	BNT162b2	8	No	Antihistamines, corticosteroids	10
23	F, 37	‐	No	‐	mRNA‐1273	10	No	Antihistamines, corticosteroids	10

Abbreviations: AD, atopic dermatitis; F, female; M, male; RC, rhino‐conjunctivitis.

To the best of our knowledge, this is the first case series of patients with delayed urticaria after the third dose of mRNA COVID19 vaccination. In literature, more than 70% of cases of delayed urticaria to COVID19 vaccines were reported after the first vaccine dose, the remaining after the second one.^3^, ^4^, ^5^, ^6^, ^7^ Accordingly to literature data, females resulted more frequently involved,^3^, ^4^, ^5^, ^6^ while our patients were younger than those reported in literature (fourth decade, mean age: 41.9 years).^3^, ^4^, ^6^, ^7^

Regarding the characteristics of vaccine‐induced delayed urticaria, latency time ranged from 7 to 11 days in 21 subjects (91.3%), longer than that reported after the first (8 h to 6 days)^4^, ^5^, ^7^ and the second (2–8 days)^3^, ^4^ vaccine doses. Urticaria was mostly severe requiring systemic corticosteroids in 13, confirming literature data.^3^, ^6^

Immunological mechanism of delayed urticaria is not clearly understood. The long latency time and the absence of systemic symptoms leading to anaphylaxis seem to exclude an IgE‐mediated hypersensitivity reaction.^4^ The short latency time observed in delayed urticaria after the first vaccine dose would seem to exclude a T‐cell mediated mechanism.^3^, ^4^ Moreover, only the minority of patients (about 40%) developed recurrent urticaria after the second vaccine dose, leading to exclude an allergy and suggesting a host immune response to the viral mRNA protein product through a potent T and B cell activation,^4^, ^6^ with rapid increase of plasma cells, memory B cells, and antibody levels, together with a strong IL‐4 production demonstrated after COVID19 vaccine BNT162b1.^9^ In conclusion, delayed urticaria may be observed not only after the first and the second mRNA COVID19 vaccine doses^3^, ^4^, ^5^, ^6^, ^7^ but also solely after the third one. More studies on larger study groups are needed to better characterize prevalence, clinical course, and management of delayed urticaria after COVID19 vaccine doses.

## AUTHOR CONTRIBUTIONS

**Leonardo Bianchi, Katharina Hansel, Luca Stingeni:** conceptualization; **Leonardo Bianchi, Filippo Biondi:** methodology; **Leonardo Bianchi, Katharina Hansel, Maddalena Napolitano:** formal analysis; **Cataldo Patruno, Luca Penchini:** data curation; **Leonardo Bianchi, Katharina Hansel, Luca Stingeni:** writing—original draft preparation. **Maddalena Napolitano, Cataldo Patruno, Gabriella Fabbrocini:** writing—review and editing**. Filippo Biondi, Luca Penchini:** visualization**. Luca Stingeni, Katharina Hansel**: supervision. All authors have read and agreed to the published version of the manuscript.

## CONFLICT OF INTEREST

The authors declare no conflict of interest.

## ETHICS STATEMENT

The study has been approved by the local ethic and research committees of the participating centers and written informed consent was provided by patients to the data publication.

## Data Availability

The data that support the findings of this study are available from the corresponding author upon reasonable request.
